# 2D-MoS_2_ nanosheets as effective hole transport materials for colloidal PbS quantum dot solar cells†

**DOI:** 10.1039/c8na00272j

**Published:** 2019-01-07

**Authors:** Srikanth Reddy Tulsani, Arup K. Rath, Dattatray J. Late

**Affiliations:** Physical and Materials Chemistry Division, CSIR-National Chemical Laboratory Dr Homi Bhabha Road Pune 411008 India dj.late@ncl.res.in datta099@gmail.com; Academy of Scientific and Innovative Research (AcSIR) Ghaziabad-201002 India

## Abstract

Herein, we demonstrate for the first time matrix-free deposition of two dimensional (2D) MoS_2_ nanosheets as an efficient hole transport layer (HTL) for colloidal lead sulfide (PbS) quantum dot (QD) solar cells. We have developed all-solution-processed n–p–p^+^ architecture solar cells where ZnO nanoparticles were used as an n-type window layer, a PbS QD layer acted as a light absorbing p-type layer and 2D-MoS_2_ nanosheets acted as a p^+^-type hole transport layer. The MoS_2_ nanosheets allow better interface with the PbS QD layers. The incorporation of the MoS_2_ hole transport layer leads to superior fill factor, higher open circuit voltage and better performance in colloidal PbS QD solar cells. These results show that one layer of MoS_2_ nanosheets improves the power conversion efficiency of the device from 0.92% for a hole transport material free device to 2.48%. The present work reveals the development of 2D-MoS_2_ nanosheets as a new hole transport layer for the fabrication of cost-effective, durable and efficient colloidal PbS quantum dot solar cells.

## Abstract 2

Herein, we demonstrate for the first time matrix-free deposition of two dimensional (2D) MoS_2_ nanosheets as an efficient hole transport layer (HTL) for colloidal lead sulfide (PbS) quantum dot (QD) solar cells.

## Graphical abstract

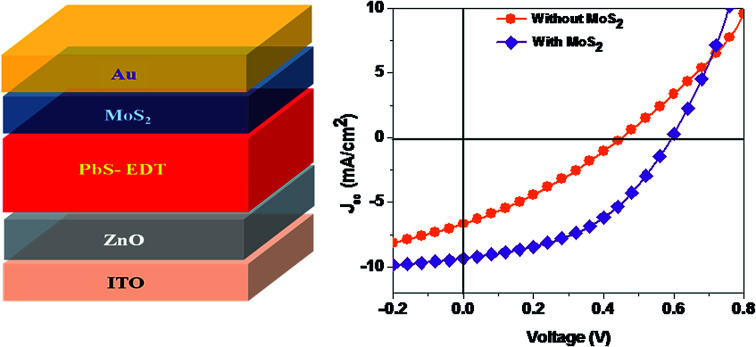

## 1 Introduction

In recent years two-dimensional (2D) transition metal dichalcogenides (TMDs) have attracted widespread attention because of their potential application in optoelectronic devices,^1^ catalysis,^2^ transistors,^3–5^ photonics,^6–9^ photodetectors,^10^ electronics,^11^ sensors,^12,13^ memories,^14,15^ and photocatalyzed hydrogen evolution reactions.^16–18^ Among all TMDs, 2D MoS_2_ attracted significant interest due to its unique graphene like properties along with remarkable optoelectronic properties, like variable energy band gap, high conductivity, good flexibility, high transparency, high surface area, *etc.* In comparison to monolayer graphene,^19^ which is semi-metallic, monolayer MoS_2_ shows semiconducting characteristics with a direct band gap. Furthermore, the thickness dependent band gap changing from an indirect band gap in bulk to a direct bandgap in monolayer MoS_2_ has opened up several applications.^20^

The development of 2D TMD-based solar cell devices is, however, limited due to the difficulties in fabricating large-scale defect-free 2D TMD materials. Even though the nanosheets, nanoparticles and bulk forms of TMD materials have been used in solar cell devices,^21–23^ they have not yet been explored in colloidal quantum dot based solar cells. For example, Shanmugan *et al.* have fabricated 220 nm thick MoS_2_ nano-membranes stacked with Au and achieved a solar cell efficiency of 1.8% by making a Schottky junction at the MoS_2_/Au interface.^21^ The bulk hetero-junction MoS_2_/TiO_2_ nano-composite material was used in solar cells, which showed a power conversion efficiency of 1.3%.^22^ Further, MoS_2_/graphene and WS_2_/MoS_2_ based atomically thin solar cell devices have been reported with exceptionally large power densities per kilogram (450–1800 kW kg^−1^).^24,25^ However, due to the high transmission loss in these monolayer-based devices, the efficiencies of WS_2_/MoS_2_ and MoS_2_/graphene based solar cells remained below 2%.

For the commercial viability of solar cell devices, it is of great importance to develop materials with ease of synthesis, cost-effective device building and high throughput performance. This can be achieved by either further improvement in the solar cell efficiency^26,27^ or reducing the cost^28,29^ of the devices or both. Silicon based solar cells have dominated the photovoltaic industry due to their abundance, high performance and mature processing technology.^26^ Therefore, in addition to exploring 2D materials, it will be of great interest to investigate their capability for solar energy conversion applications. To reach the goal, wafer-scale fabrication methods for 2D materials have recently been implemented.^30–32^ To date, devices based on large-area multilayer TMD materials have not yet been widely established due to the stringent growth conditions of 2D TMD materials.

Here for the first time, we report MoS_2_ nanosheets as a hole transport material for colloidal PbS quantum dot solar cells. The surface of the MoS_2_ nanosheets is functionalized with molecular iodine to disperse them in DMF. This allows the matrix free deposition of MoS_2_ on top of the PbS QD layer for hole transport layer formation. The valence band position of the MoS_2_ layer matches well with that of PbS QDs for efficient hole transfer, which has been exploited to improve the photovoltaic performance of solar cell devices.

## 2 Experimental section

### Chemicals used

Sodium molybdate dihydrate (>99.0%), sulfur powder, sodium borohydride (>98.0%), *N*,*N*-dimethylformamide (99.8%), tetrabutylammonium iodide (TBAI) (98.0%), lead oxide (99.0%), 1-octadecene (90%), oleic acid (90%), bis(trimethylsilyl) sulfide (TMS), zinc acetate trihydrate (99.8%), 2-methoxyethanol (99.9%), ethanolamine(99%), 1,2-ethanedithiol (>98.0%) (EDT), acetone, methanol and toluene were purchased from Sigma-Aldrich. Solvents used in reactions are all anhydrous. The DI water used had a resistivity of 18.2 million ohm cm.

### Synthesis of MoS_2_ nanosheets

The MoS_2_ nanosheets were synthesized by using a facile hydrothermal route.^33^ Briefly, in this synthesis process, 0.56 grams of Na_2_MoO_4_, 0.25 grams of sulphur powder and 0.1 gram of NaBH_4_ were weighed, dissolved in 40 mL of DI water and stirred for half an hour to achieve proper dispersion in DI water. Then the reaction mixture was transferred into a Teflon lined stainless steel autoclave and kept at 200 °C for 24 hours. After cooling down to room temperature, the product was cleaned with DI water twice, followed by washing with ethanol twice, and then it was centrifuged at 5500 rpm. Finally, the product was dried in a vacuum oven to remove the residual solvents from the material. The as-synthesized MoS_2_ material was taken in 3 mL DMF solvent. 200 mg TBAI was added to the solution and the mixture was heated at 100 °C under continuous stirring for 12 hours under an Ar atmosphere. Iodine functionalized MoS_2_ nanosheets were then isolated by addition of toluene, followed by centrifugation. The dry MoS_2_ powder was then dispersed in DMF at a concentration of 20 mg mL^−1^ for film fabrication.

### PbS QD synthesis

The PbS QDs were synthesized by using the hot injection method.^34^ Briefly, 0.45 grams of lead oxide, 3 mL octadecene (degassed under vacuum for 12 hours before using) and 1.5 mL oleic acid were mixed in a three neck round bottom (RB) flask and kept in a vacuum at 95 °C using a standard Schlenk line for 12 hours. The reaction mixture turned into a clear solution upon the formation of the lead oleate complex. The reaction with vacuum (10^−2^ mbar) environment was then purged with argon gas. Then 15 mL octadecene was added to this reaction mixture and the temperature was set to 120 °C. At this temperature, 210 μL TMS dissolved in 10 mL octadecene was then injected quickly into the reaction bath without delay and the entire solution turned dark brown immediately, indicating PbS nucleation. The heating source was switched off and the reaction bath was allowed to cool down naturally to room temperature. After reaching room temperature, 24 mL of acetone was then added to the reaction bath and the mixture was allowed to stir for 3 minutes. The PbS QDs were isolated by centrifugation at 3500 rpm for 5 minutes. To clean the QDs they were dispersed in a small amount (∼3 mL) of toluene, precipitated with 24 mL of acetone, followed by centrifugation, and finally dispersed in toluene.

### ZnO sol–gel synthesis

The zinc oxide sol–gel solution was prepared by dissolving 1 gram of zinc acetate, 10 mL 2-methoxyethanol and 284 microliters of ethanolamine added in the same sequence at room temperature.^35^ The precursor solution was continuously stirred overnight at room temperature to induce hydrolysis in the presence of air. The solution was filtered through a 0.2 μm syringe filter for further use in device fabrication.

### ZnO film deposition

Commercially available indium tin oxide (ITO) coated glass substrates were first etched by using hydrochloric acid and zinc dust. After etching, they were sonicated in a soap solution, DI water, acetone and isopropanol for 30 minutes each and finally the substrates were cleaned in boiling isopropanol for 15 minutes. Then the ZnO sol–gel filtered solution was spin coated on the ITO substrates at a rotation speed of 3500 rpm for 30 s. The as-deposited films were then annealed at 200 °C for 10 minutes. The substrates were then coated with another layer of ZnO under the same spinning conditions and annealed at 260 °C for 30 minutes.

### Solar cell device fabrication

As prepared above ZnO substrates were then used for PbS QD deposition. PbS QDs were deposited *via* a layer by layer (LBL) solid state ligand exchange approach under ambient conditions.^36^ 1,2-Ethanedithiol (EDT) 2% v/v in acetonitrile solution was used as a ligand for the deposition of PbS QDs. The film deposition steps followed in this work were as follows: PbS QDs (30 mg mL^−1^ in toluene) were first spin coated at 2500 rpm on the substrates for 30 seconds. Under continuous spinning 4 drops of EDT were added, followed by rinsing with 8 drops of acetonitrile to remove excess EDT. This completes one layer of PbS QD film deposition. The sequence was repeated to grow layer by layer films up to 8 layers. On top of the PbS QD films, one layer of MoS_2_ (20 mg ml^−1^) was deposited by spin coating at a speed of 2500 rpm for 40 s. Top electrodes were deposited using a thermal evaporator (Hind High Vacuum, model BC-300) at a base pressure of 3 × 10^−6^ mbar. 10 nm MoO_3_ was deposited at 0.1 Å s^−1^, followed by 30 nm Au deposition at 0.5 Å s^−1^, and finally, 120 nm Ag was deposited at 1 Å s^−1^ to complete the solar cell device fabrication.

### Current–voltage measurement

The current–voltage (*I*–*V*) measurement was carried out using a Keithley 2634B source-meter at ambient room temperature. The light intensity of AM1.5 was provided using a solar simulator (class-AAA) from Peccell Technologies (PEC-L01). A shadow mask was used to match the illuminated area with the device area. Using a Thorlabs flat band thermal sensor S302C (aperture size 9.3 mm), the light intensity was set at 100 mW cm^−2^.

#### Morphological and structural analysis

The as-synthesized MoS_2_ nanosheet powder sample was characterized by X-ray diffraction (XRD) on a PANalytical X'pert Pro diffractometer using Cu Kα (1.5418 Å) radiation. Microscopic studies of the synthesized MoS_2_ nanosheet powder samples were carried out using a transmission electron microscope (TEM) (FEI model TECNAI F20 S/TEM) and a high resolution transmission electron microscope (HR-TEM) (JEOL model JEM-F200). Samples for TEM and HR-TEM analysis were prepared by dispersing a small amount of the sample in ethanol and dropped on to a carbon coated copper grid (Type-B 200 mesh). The cross-sectional image of the device was analyzed using a field emission scanning electron microscope (FE-SEM) (FEI model Nova NanoSEM 450). The morphology of MoS_2_ nanosheets was analyzed using an environmental scanning electron microscope (E-SEM) (FEI model Quanta 200 3D). The Raman spectra were measured with a micro-Raman spectrometer (Horiba JY Lab Raman HR 800) at an excitation laser wavelength of 632.8 nm. For AFM analysis of MoS_2_ nanosheets, the atomically thin MoS_2_ nanosheets dispersed in ethanol were deposited at room temperature under ambient conditions on Si substrates. The thinnest MoS_2_ sheets were identified by optical microscopy. The MoS_2_ sheets had typical lateral dimensions of few micrometers. The as-synthesized MoS_2_ sheets were characterized by AFM (Nanosurf).

## 3 Results and discussion

**Fig. 1:**
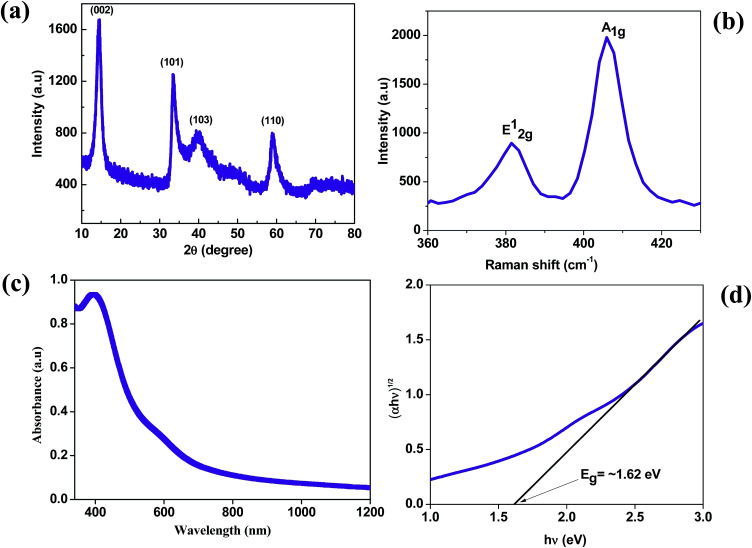
(a) X-ray diffraction pattern of MoS_2_. (b) Raman spectrum of MoS_2_. (c) Absorption spectrum and (d) Tauc plot of MoS_2_.

The MoS_2_ nanosheets synthesized by the hydrothermal method were analyzed by X-ray diffraction (XRD) to confirm their structure, phase and crystallinity. The XRD pattern of the MoS_2_ nanosheets is shown in Fig. 1(a). The MoS_2_ nanosheets showed a strong diffraction peak at 14° which corresponds to the (002) plane with a *d*-spacing of 0.62 nm, which is indexed to a hexagonal phase of MoS_2_ (JCPDS no. 37-1492), and showed a well-stacked layered structure along the *x*-axis.^33^ The Raman spectrum of the MoS_2_ nanosheets is shown in Fig. 1b. The MoS_2_ nanosheets showed two prominent Raman bands at ∼381.3 cm^−1^ and ∼405.8 cm^−1^, which are ascribed to the E^1^_2g_ and A_1g_ vibrational modes, respectively.^37^ The out-of-plane A_1g_ mode indicates the symmetric vibration of S atoms along the *c*-axis and the in-plane E^1^_2g_ mode indicates the opposite vibration of two S atoms relative to the Mo atom. The Raman frequencies of A_1g_ and E^1^_2g_ peaks change with the number of layers in the MoS_2_ nanosheets, and the frequency difference between A_1g_ and E^1^_2g_ modes is 24.5 cm^−1^ which corresponds to the multilayer nature of the as-synthesized MoS_2_ nanosheets.^38^

To estimate the band gap of the as-synthesized MoS_2_ nanosheets, we measured the absorption spectra as shown in Fig. 1(c). The small noticeable peak at ∼600 nm corresponds to the spin–orbit split pair originating from the band edge excitonic transitions at the *K* point of the Brillouin zone.^39^ The broad peak at ∼395 nm corresponds to interband transitions from the occupied d_*z*^2^_ orbital to unoccupied d_*xy*_, d_*yz*_, d_*xz*_ and d_*x*^2^–*y*^2^_ orbitals.^40^ From the absorption spectra, the calculated optical band gap is found to be ∼1.62 eV by using the Tauc plot with the allowed transitions for the MoS_2_ nanosheets.

**Fig. 2:**
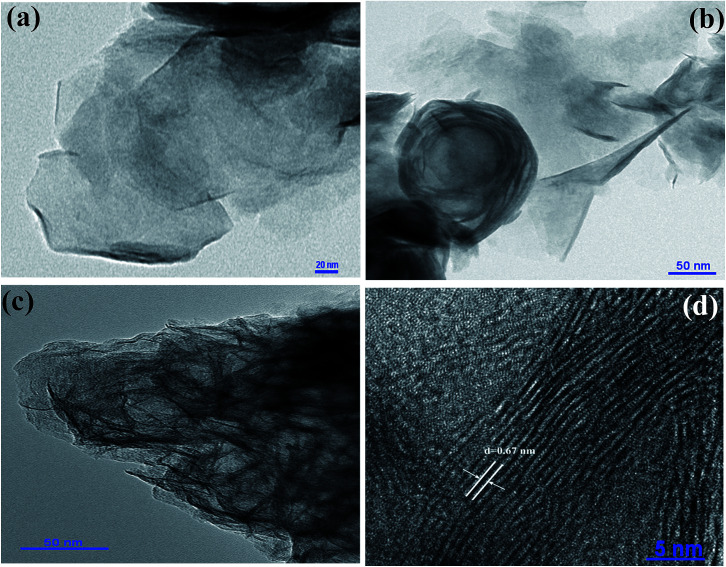
(a)–(d) show the TEM images of MoS_2_. *d*-Spacing between the MoS_2_ layers shown in figure (d) is 0.67 nm.

Fig. S1 (a and b)† shows the typical scanning electron microscopy (SEM) images of the MoS_2_, indicating the nanosheet morphology of the as-synthesized material of few micrometers in length. High-resolution transmission electron microscopy (HR-TEM) was used for further analysis of MoS_2_ nanosheets. Fig. 2 (a–d) show the high-resolution TEM images of MoS_2_ nanosheets; all the TEM images suggest a nanosheet-like morphology, consistent with SEM images. The folded edges show parallel lines in Fig. 2(d), corresponding to different layers of MoS_2_ sheets with a *d*-spacing of ∼0.67 nm. The corresponding selected area electron diffraction (SAED) patterns shown in Fig. S2† indicate the crystalline nature of MoS_2_ nanosheets which supports the XRD and high-resolution TEM results. Further, energy dispersive X-ray analysis (EDAX) of MoS_2_ nanosheets was carried out. EDAX data are shown in ESI Fig. S3,† showing the stoichiometric ratios between Mo and S. Absorption spectra of oleic acid capped PbS quantum dots dispersed in toluene are shown in ESI Fig. S4.† The TEM images of PbS quantum dots are shown in Fig. S5,† indicating monodispersed QDs with an average size of ∼3.2 nm.

**Fig. 3:**
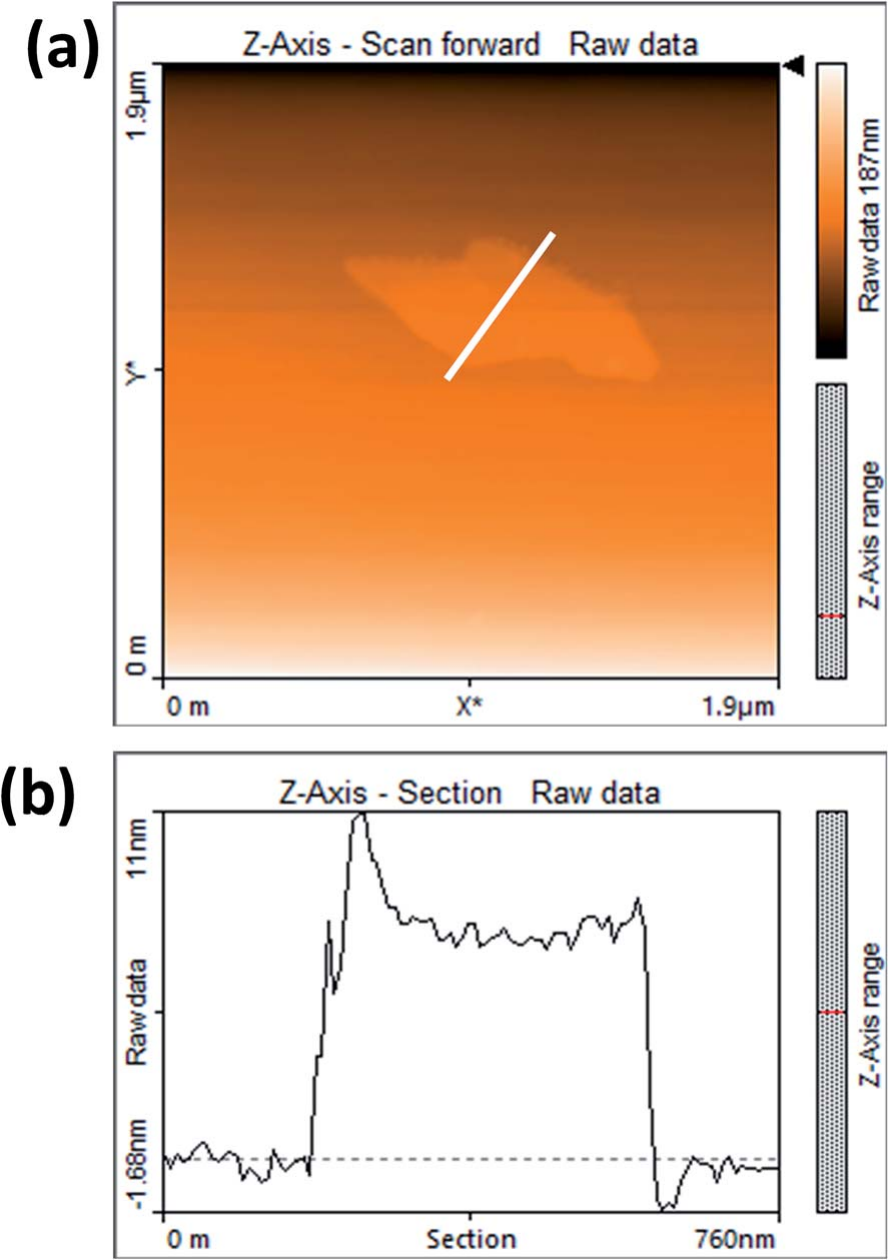
(a) AFM image of MoS_2_ nanosheets and (b) corresponding AFM height profile of MoS_2_ nanosheets.

**Fig. 4:**
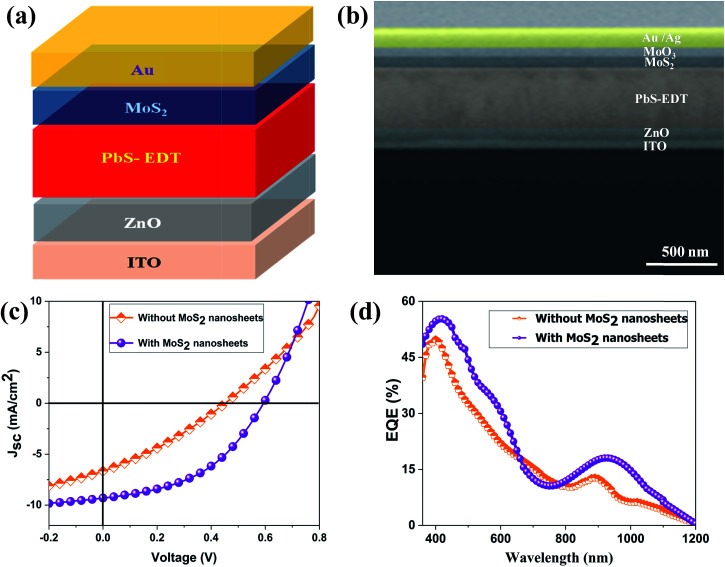
(a) Solar cell device architecture. (b) Cross sectional SEM image of the device. (c) The *I*–*V* characteristics of the solar cells. (d) External quantum efficiency (EQE) of the solar cells.

**Fig. 5:**
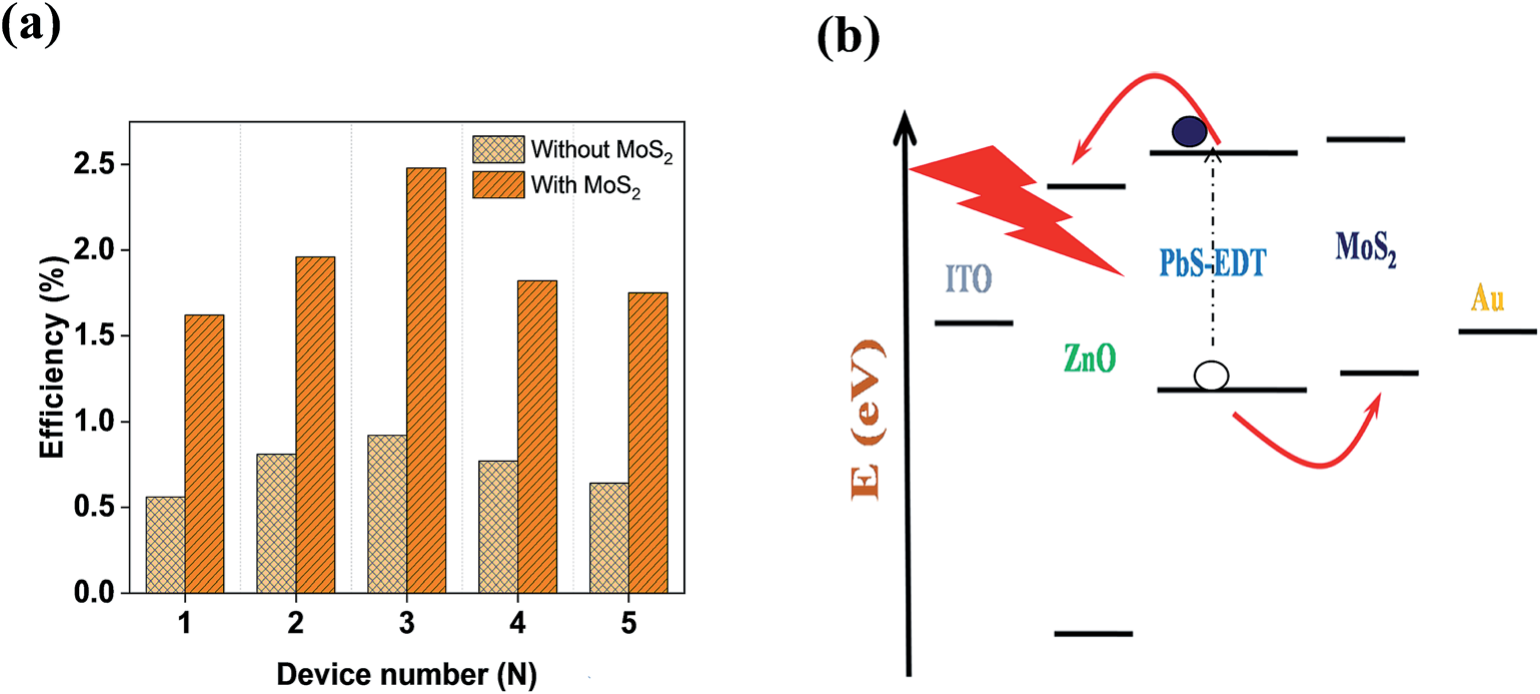
(a) Statistical variation of the solar cells. (b) Energy level diagram of a solar cell.

Fig. 3(a) shows the contact mode atomic force microscopy (AFM) topography of a thin MoS_2_ nanosheet deposited on a Si substrate. We have used contact mode AFM to determine the thickness and to characterize the topography of the deposited sheets. The height profile obtained from the solid white line is shown in Fig. 3(b). It is noted that the height of the MoS_2_ nanosheet deposited on a Si substrate is found to be ∼10 nm. The as-synthesized MoS_2_ nanosheets are used as a hole transport layer to build PbS quantum dot solar cells. The solar cell device architecture is shown in Fig. 4(a) and the corresponding cross sectional SEM image is shown in Fig. 4(b). The typical *I*–*V* characteristics for devices with and without the MoS_2_ hole transport layer are shown in Fig. 4(c). The device without MoS_2_ shows a power conversion efficiency of 0.92%, with an open circuit voltage (*V*_oc_) of 0.46 V, a short circuit current density (*J*_sc_) of 6.84 mA cm^−2^ and a fill factor of 30%. The insertion of MoS_2_ nanosheets as a hole transport layer improves the efficiency from 0.92% to 2.48% due to overall increments in *V*_oc_, *J*_sc_ and FF. The EQE results shown in Fig. 4(d) show higher photon to current conversion ratios for the entire wavelength range for the device with MoS_2_ nanosheets compared with the pristine device. The increase in EQE (%) and *J*_sc_ is attributed to superior charge collection in the latter case. The photovoltaic figures of merits of the photovoltaic devices are shown in Table 1. Fig. 5(a) shows the typical statistical variations in the efficiency of five solar cell devices. The enhancement in photovoltaic performance due to the insertion of the MoS_2_ layer is explained by the energy band diagram, shown in Fig. 5(b). In the absence of the MoS_2_ layer photo generated electrons and holes both are allowed to transfer to the Au electrode. This would enhance the photo carrier recombination at the PbS–Au interface and thereby reduce the current generation of the solar cells. Further, the 1.62 eV band gap of the MoS_2_ layer pushes its conduction band position well above the conduction band of PbS QDs, which blocks the transfer of electrons from PbS to the MoS_2_ layer. The selective extraction of holes at the PbS–MoS_2_ interface reduces the recombination of the photo generated carriers, thereby enhancing the current generation of the solar cells. Further, the hole carrier selectivity of the MoS_2_ layer reduces the voltage loss by reducing the Fermi energy pinning at the PbS–Au interface which helps to increase the voltage generation and fill factor of the solar cells.

**Table 1:** Device performance of solar cells with and without molybdenum disulfide

Device	*V*_oc_ (V)	*J*_sc_ (mA cm^−2^)	Fill factor	Efficiency (*η*%)
Without MoS_2_	0.46	6.84	0.30	0.92
With MoS_2_	0.60	9.30	0.45	2.48

## 4 Conclusions

In conclusion, we have demonstrated the matrix free deposition of 2D MoS_2_ nanosheets by the spin coating process. We have successfully developed a hole transport layer by using 2D MoS_2_ nanosheets, which improves the efficiency of PbS quantum dot solar cell devices from ∼0.92% to ∼2.48%. In solar cell devices it improves the charge extraction and reduces the carrier recombination. These improvements help to boost the efficiency of the solar cells by enhancing the short circuit current, open circuit voltage and fill factor. Our results open up new opportunities to use inorganic 2D MoS_2_ as an alternative to organic hole transporting materials for the development of the next generation solar cell devices.

## Conflicts of interest

All authors declare no conflicts of interest.

## Supplementary Material

NA-001-C8NA00272J-s001
