# Supplementary material for: 2D-MoS2 nanosheets as effective hole transport materials for colloidal PbS quantum dot solar cells
Source: Nanoscale Adv. 2019 Jan 7;1(4):1387–94. doi: 10.1039/c8na00272j (PMC9418634; doi:10.1039/c8na00272j)
Supplement: NA-001-C8NA00272J-s001 [file NA-001-C8NA00272J-s001.pdf]

Electronic Supplementary Material (ESI) for Nanoscale Advances.
This journal is © The Royal Society of Chemistry 2019

Supporting Information

2D-MoS_2 nanosheets as effective hole transport materials for colloidal PbS quantum dot solar cells

Srikanth Reddy Tulsani[1,2], Arup K.Rath[1,2], Dattatray J. Late[1,2]*

[1]Physical and Materials Chemistry Division, CSIR-National Chemical Laboratory, Dr. Homi Bhabha Road, Pune, 411008, India.

[2]Academy of Scientific and Innovative Research (AcSIR), Ghaziabad- 201002, India

Figure S1:

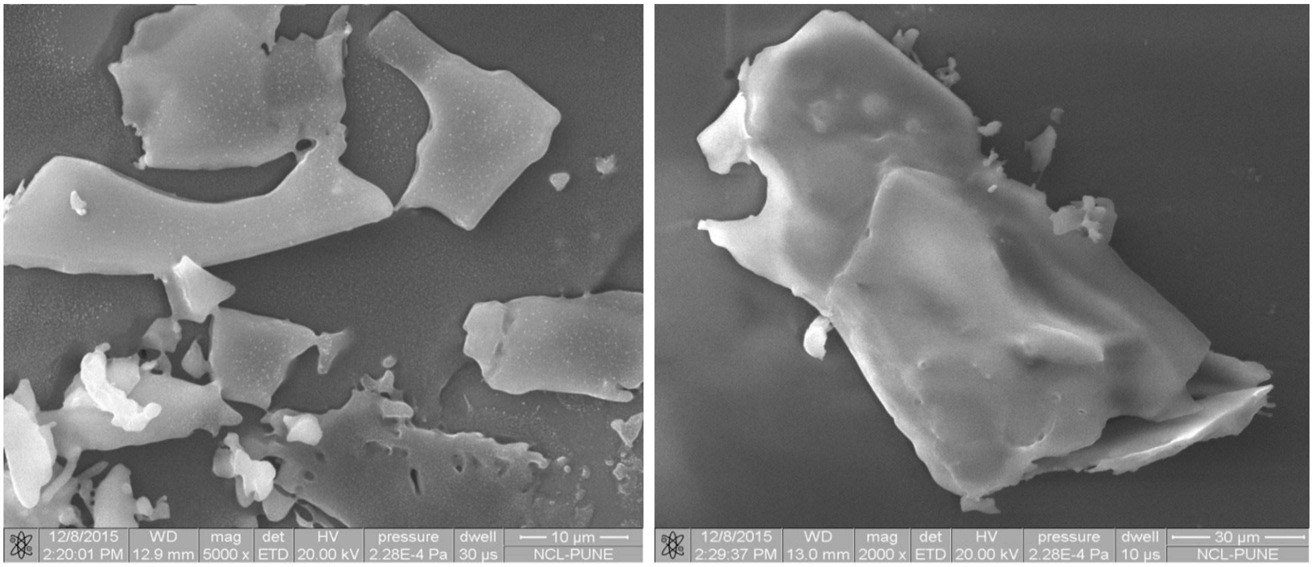

Figure S1. SEM images of MoS_2 nanosheets showing lateral dimension of the sheet between 10 to 100 μm

Figure S2:

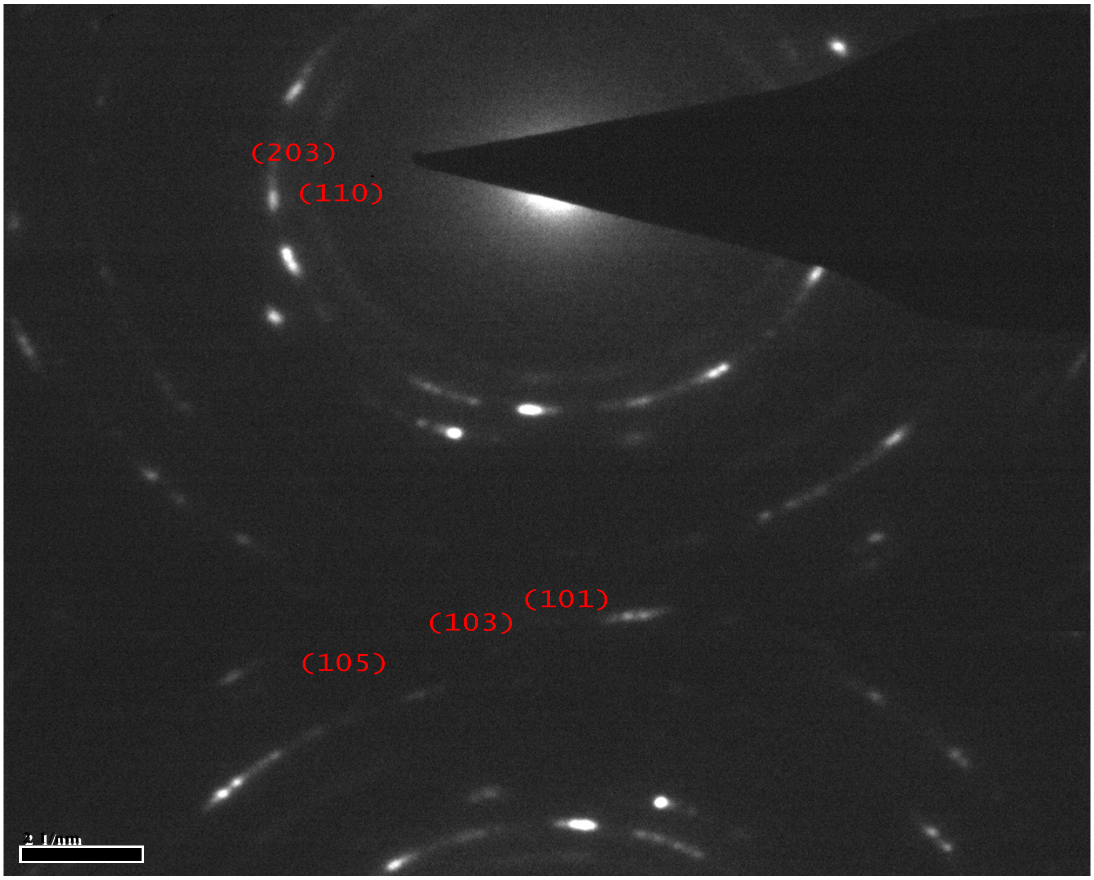

Figure S2. Typical SAED pattern of MoS_2 nanosheets.

Figure S3:

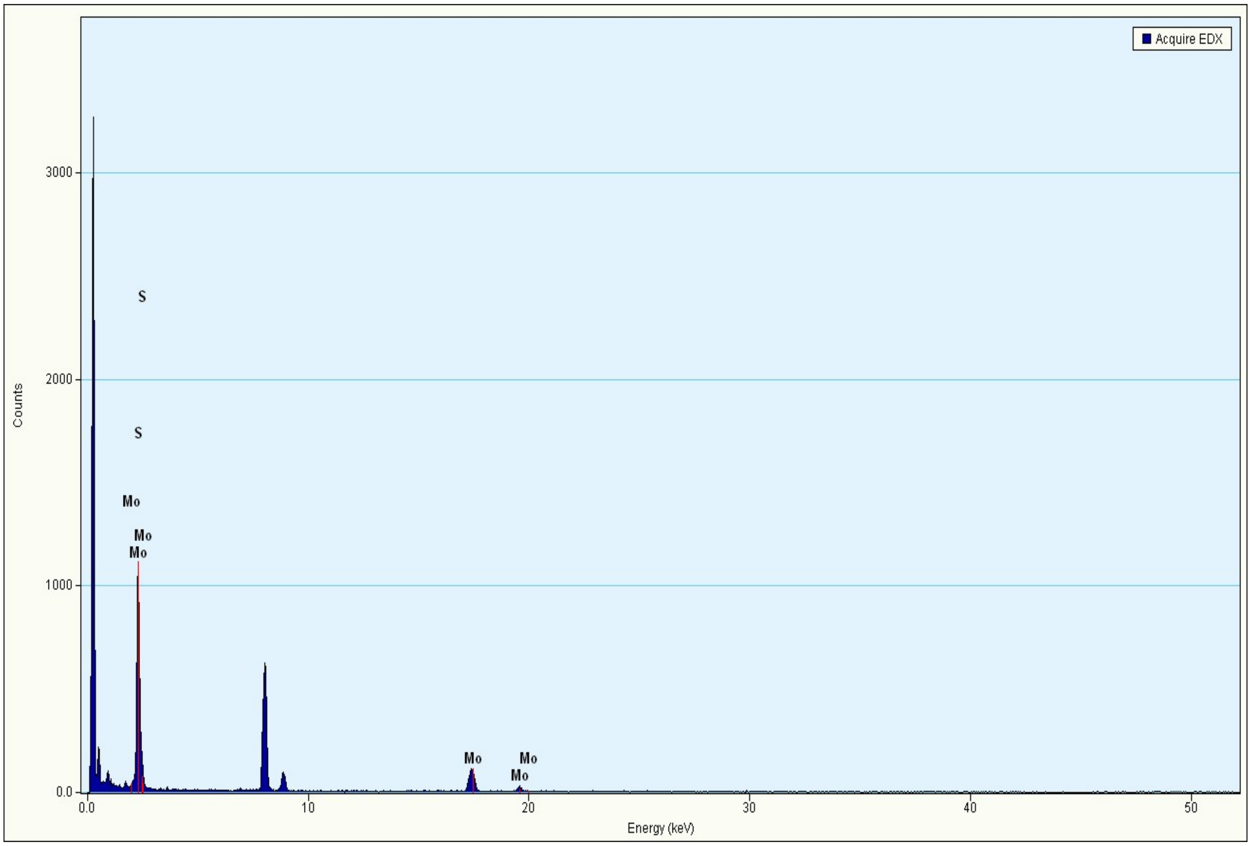

Figure S3. Energy Dispersive X-Ray Analysis (EDAX) of MoS_2 nanosheets.

Figure S4:

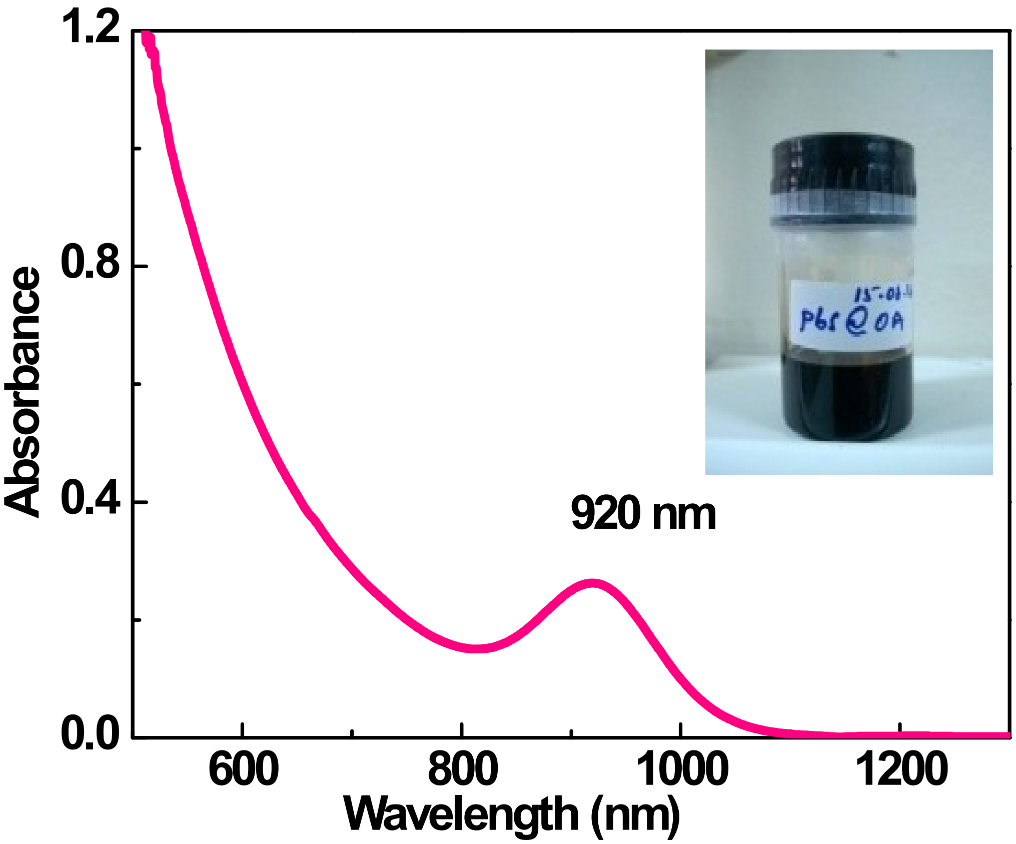

Figure S4. Absorption spectra of oleic acid capped PbS quantum dots; inset picture shows dispersed quantum dots in toluene.

Figure S5:

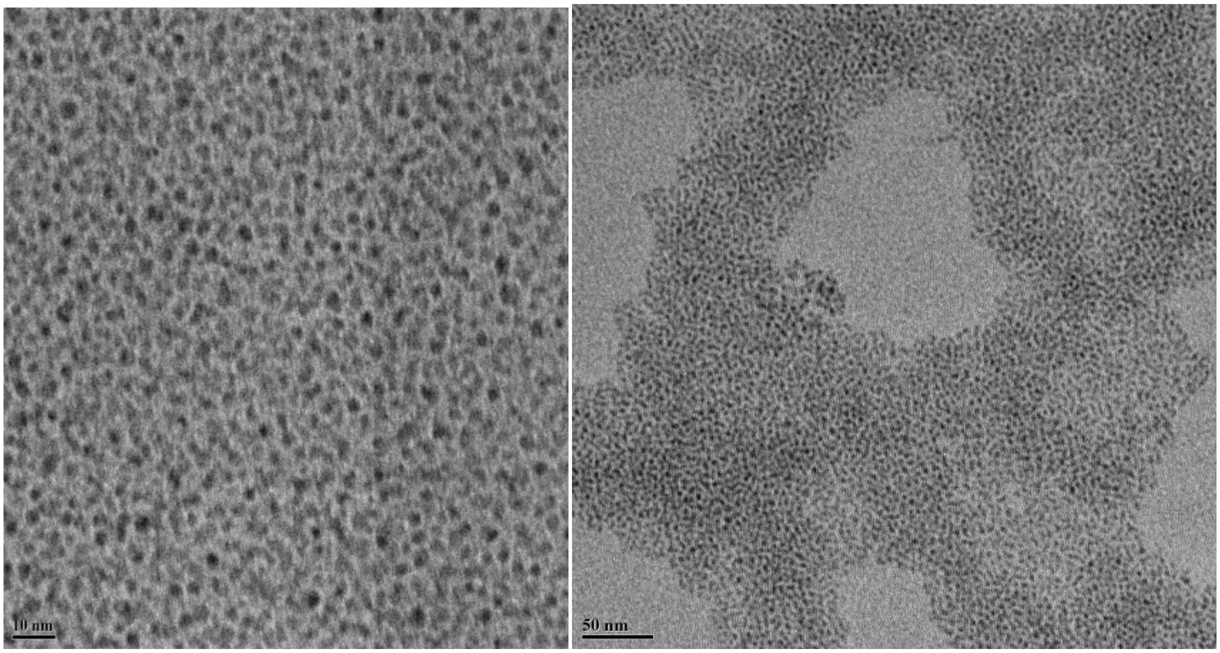

Figure S5. Typical TEM images of PbS quantum dots.
